# “We Are Humans, and We Are People” - A Thematic Analysis Exploring the Disclosure and Help-Seeking Experiences of Young People Who Experience Voice-Hearing Within Mental Health Services in the UK

**DOI:** 10.1177/13591045251400393

**Published:** 2025-11-25

**Authors:** Megan Ellis, Mark Hayward, Clio Berry, David Fowler, Aikaterini Rammou

**Affiliations:** 1 School of Psychology, 152144 University of Sussex, Brighton, UK; 2 Research & Development Department, Sussex Partnership NHS Foundation Trust, UK; 3 Department of Primary Care and Public Health, Brighton and Sussex Medical School, University of Brighton and University of Sussex, Brighton, UK

**Keywords:** auditory verbal hallucinations, voice-hearing, hearing voices, CAMHS, qualitative study, transdiagnostic, help-seeking, disclosure

## Abstract

**Background:**

Voice-hearing is increasingly being recognised as a transdiagnostic experience which is common for children and adolescents. However, little is known about how young people seek help and disclose voice-hearing within mental health services.

**Method:**

This qualitative study explored the disclosure and help-seeking experiences of nine young people (aged 14–18) receiving care from mental health services in the UK. Semi-structured interviews were conducted and analysed using thematic analysis within a critical realist framework.

**Results:**

Two superordinate themes were identified: barriers to accessing help; facilitators to accessing help; and the impact of practitioner response on young people’s engagement. Stigma, long waiting lists for services, and practitioners’ lack of knowledge often acted as barriers to disclosure and help-seeking, whereas trust and clear communication facilitated disclosure and engagement. Participants often wished to be listened to, to be offered a more personalised approach and greater flexibility from mental health services. When practitioners demonstrated empathy and allowed trust to build in the therapeutic relationship, participants felt valued.

**Conclusions:**

Findings suggest that practitioners might need to be supported to build confidence in discussing voice-hearing with young people to facilitate therapeutic conversations about these experiences, and that offering flexible, person-centred support may support young people’s engagement with mental health services.

## Plain Language Summary

**What was this research about?** Some young people hear voices that others don’t, and for many, this can be distressing. Although fairly common in children and teenagers, little is known about how young people talk about hearing voices or seek support, especially within mental health services. Understanding their experiences can help improve how services respond. **What did the researchers do?** A researcher interviewed nine young people aged 14 to 18 who were receiving care from UK mental health services and had experience of hearing distressing voices. They were asked about who they shared this with and what helped or made it harder to get support. The research team analysed their stories to identify common themes. **What did the researchers find?** Young people often delayed talking about hearing voices because they were worried about being judged or misunderstood. Some avoided services or found it hard to trust professionals. Barriers included long waiting times, being passed between services, and professionals not knowing much about voice-hearing. Participants felt frustrated when their experiences were dismissed or avoided. On the other hand, young people felt more comfortable opening up when practitioners built trust, listened without judgement, and explained available support. Flexible, personalised care was valued over rigid, one-size-fits-all approaches. Feeling accepted and understood helped young people feel more confident in managing their experiences. **Why is this important? **The findings suggest mental health services could better support young people who hear voices by offering flexible care, building trust, and ensuring practitioners are trained and informed. These changes could help reduce stigma and encourage more young people to seek help and get the support they need.

## Background

Voice-hearing, also known as auditory verbal hallucinations, is most commonly understood as hearing a voice or voices that no-one else can hear in the absence of any clear auditory stimuli. This experience has historically been associated with disorders on the schizophrenia spectrum (Moseley et al., 2013) but is increasingly being recognised as a transdiagnostic experience, existing in non-clinical samples and across mental health diagnoses (Pierre, 2010; Preti et al., 2014; Waters et al., 2018), including affective disorders such as bipolar and unipolar depression (Baethge et al., 2005; Shinn et al., 2012), borderline personality disorder (Slotema et al., 2012) and trauma-spectrum disorders such as posttraumatic stress disorder and dissociative disorders (Shinn et al., 2020).

Voice-hearing is also an experience commonly reported by young people (Maijer et al., 2019), with prevalence estimations ranging from 5% to 35% (Bartels-Velthuis et al., 2010; Dhossche et al., 2002; Maijer et al., 2017, 2018). Furthermore, a recent meta-analysis highlighted the higher prevalence of voice-hearing in young people than adults (Maijer et al., 2018). However, whilst voice-hearing is a common experience in young people, the experience is often transient (Bartels-Velthuis et al., 2016) and this has led to a recommendation that intervention for those seeking help should follow a ‘curious but cautious’ approach, with a focus upon the factors that may be maintaining voice-hearing (Maijer et al., 2019).

## Disclosure of Voice-Hearing

The decision to disclose voice-hearing can be complex and there is evidence that individuals may be reluctant to disclose at initial onset (Milligan et al., 2013). Some individuals may feel unconcerned by their experience and only disclose it when they feel they are struggling to cope (Yttri et al., 2020). Others may view voice-hearing as a positive extension of the self, offering companionship or guidance (Valavanis et al., 2019). When interviewing young adults about their experience of disclosing distressing voice-hearing in Early Intervention in Psychosis Services (EIPS), Bogen-Johnston et al. (2019) reported that stigma and perceived social impact influenced disclosure decisions. Additionally, Yttri et al. (2020) reported long periods of time between experiencing voice-hearing and initial disclosure, with initial disclosure often taking place within mental health services rather than to trusted individuals.

Stigma surrounding voice-hearing experiences can be reinforced by pathologising and misleading portrayals documented by news media outlets, affecting societal stigma and the internalised beliefs of those who experience voice-hearing (Vilhauer, 2015). Following the growth of social media and smartphone use by young people (Ofcom, 2023), these portrayals may have a particularly strong impact on this population. Considering mental health difficulties more broadly, young people have reported concerns about disclosure due to fears it may jeopardise their social status among peers (Gronholm et al., 2016) or lead to negative reactions from others (Anglin et al., 2014; Yang et al., 2015). Specifically regarding voice-hearing, stigma-related concerns have been linked to reluctance to disclose, with some young people fearing unpleasant responses from others that could result in social withdrawal (Parry et al., 2021). While Parry et al. (2021) represents one of the few studies exploring this issue in young people, most existing research has focused on adults, including that by Bogen-Johnston et al. (2019) and Yttri et al. (2020). Thus, a notable gap remains in the literature concerning how young people under 18 years of age experience disclosure of voice-hearing, particularly those accessing secondary mental health services, e.g. Child and Adolescent Mental Health Services (CAMHS) or Early Intervention in Psychosis Services (EIPS), a specialist mental health service that provides early assessment and treatment for individuals experiencing a first episode of psychosis, typically including those aged 14 and above.

### Help-Seeking for Voice-Hearing

Help-seeking for any mental health problem can be challenging due to stigmatising attitudes and internalised stigma (Hellström & Beckman, 2021). The same is true when help-seeking for distressing voice-hearing. When accessing help, young people have been shown to value peer-support groups, highlighting the importance of a safe, destigmatising environment in which to discuss experiences (Newton et al., 2007). Kapur et al. (2014) investigated the experiences of young people with voice-hearing and their families when accessing mental health services such as CAMHS. They reported that over half of participants found accessing help challenging, with parents wishing for a more holistic approach from mental health services. Young people felt as though they were not listened to, professionals did not consider voice-hearing, and they wished for normalisation of these experiences. Participants also found the lack of information on voice-hearing challenging as it impacted their understanding. These findings were recently corroborated by the results of an extensive survey of CAMHS practitioners who reported only moderate levels of confidence in providing useful information to young people who were distressed by voice-hearing (Rammou et al., 2023a).

### Aims of the Current Study

There is a lack of research on the experiences of help-seeking young people who are distressed by voice-hearing, particularly outside of a psychosis context. Whilst voice-hearing experiences can be associated with a range of multi-modal hallucinations, e.g., visions, this study adopts a focus on auditory experiences. The aim of this study was to investigate the disclosure and help-seeking experiences of young people who sought help for distressing voice-hearing from CAMHS and EIPS within the UK’s National Health Service. This led to the following research question:

How do young people experience the disclosure of voice-hearing and seeking help for these experiences within mental health services?

This question reflects the conceptual intersection between disclosure and help-seeking, as disclosure is often a prerequisite for accessing support. At the same time, it allows for an examination of the distinct processes involved within each experience.

## Method

### Design

The present study was part of a larger project on factors associated with distressing voice-hearing experiences in youth (Rammou et al., 2022; 2023b). For this study a qualitative design was employed considering a subsample of the participants who had previous or were having current voice-hearing experiences. The research team conducted semi-structured interviews with participants. Following completion of the interviews, the data were transcribed verbatim and anonymised. Semi-structured interviews allowed for specific questions to be asked, maintaining a focus on the literature gap, whilst also allowing participants flexibility in their responses. This approach was particularly suited to capturing retrospective reflections on help-seeking and disclosure experiences, that were central to the study’s aims.

### Participants

Nine participants were recruited from CAMHS and EIPS within an NHS Foundation Trust in the South of England. Participants met the following eligibility criteria:

A. Aged 14 to 18 years old
B. Had previous voice-hearing experience for longer than one month
C. Had taken part in a previous quantitative study led by the research team and had agreed to be contacted for an interview
D. Were under the care of CAMHS or EIPS

These criteria were based on the design of the original quantitative study from which participants were drawn, which used measures validated for adolescents and required a minimum of one month of voice-hearing experience to ensure participants had sufficient opportunity for meaningful reflection. There were no eligibility criteria based on diagnosis as this study adopted a transdiagnostic approach consistent with emerging research indicating that voice-hearing is not confined to specific diagnoses (Waters & Fernyhough, 2017). All participants will be referred to by pseudonyms throughout the entirety of this report.

**Figure 1.**
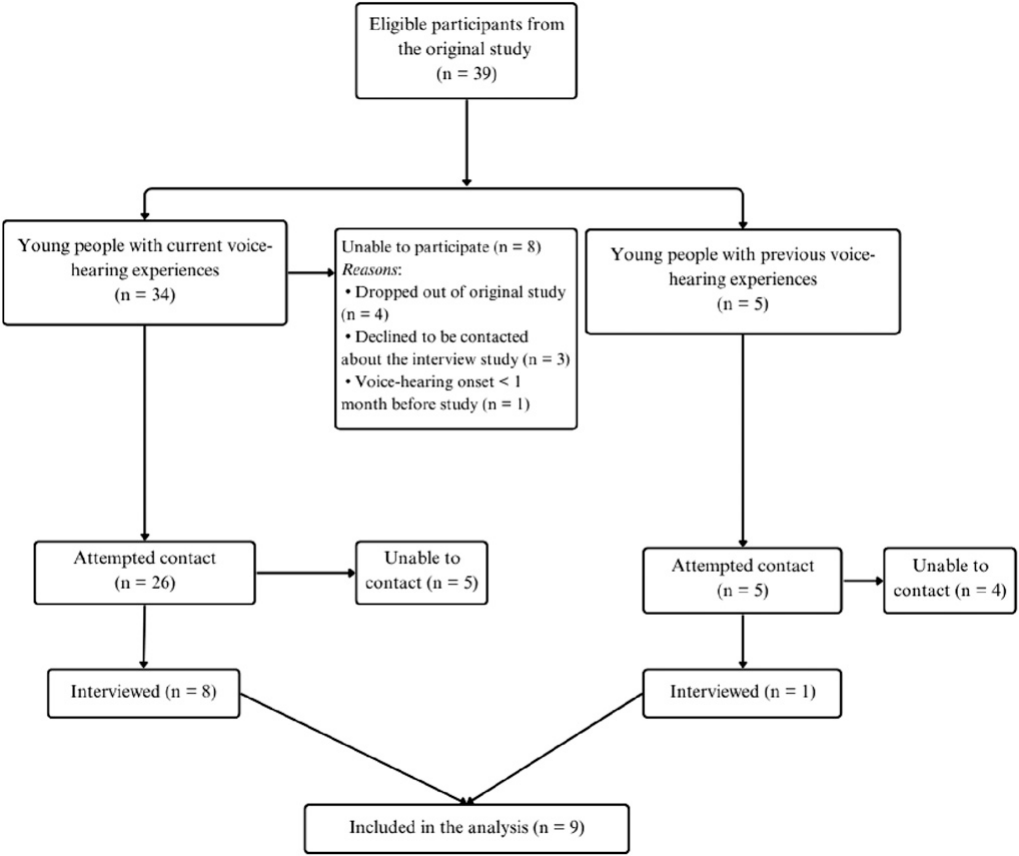
A CONSORT Diagram Showing the Flow of Eligible Participants From the Original Study Through to the Present Qualitative Follow-Up Study

The full sampling method is presented in Figure 1.

### Materials

Interviews were conducted in person and were audio recorded using a dictaphone. A semi-structured interview schedule was developed by the research team to guide interviews with participants. This included prompts under the following headings: general questions about voice-hearing experience, relating to the voices, disclosure, help-seeking, care pathways in the NHS, barriers to accessing support, practitioners’ response to disclosure, practitioners’ immediate support, long term support, positive aspects of current support, additional support suggestions and psychological therapy and ending. The full interview schedule can be found in the Supplementary Material.

### Procedure

As part of the larger project on distressing voice-hearing experiences in youth (Rammou et al., 2022; 2023b), participants had previously provided written consent to be contacted for a follow-up interview. Ethical and Health Research Authority approval was obtained through London - Brighton & Sussex Research Ethics Committee (reference number: 17/LO/2078). Following contact, participants were given a Participant Information Sheet to read, and an interview time and location were then arranged. Interviews were conducted during usual working hours (9am–5pm) at various assessment centres or at participants’ homes to minimise travel-related barriers. Participants were also offered the option of bringing a trusted friend or family member for support. At the interview, the Participant Information Sheet was reviewed again, and informed consent forms were completed. For those under 16 years of age, consent was also obtained from an adult with parental responsibility. Interviews were conducted by the last author between July 2018 and May 2019, with durations ranging from 52 minutes to 1 hour and 45 minutes.

### Analysis

Anonymised transcripts were used for analysis and NVivo was used for all data management. A thematic analysis (TA) methodology was utilised for data analysis following Braun and Clarke’s (Braun & Clarke, 2006, 2022) six phases of analysis. This enabled an in-depth understanding of participants’ help-seeking and disclosure experiences within an exploratory approach. Data analysis was conducted by the first and second authors, working in close collaboration with the last author. A critical realism position was taken during analysis with a contextual epistemological standpoint. This was due to researcher belief that the knowledge gained when learning from lived experiences might enact change processes within mental health services. A contextual approach allows for focus to be on the participant’s experiences of treatment and accepts that knowledge is truthful in certain contexts whilst also accepting there is no single reality and knowledge is temporary, changing as perspectives change (Braun & Clarke, 2013).

During familiarisation, each transcript was read three times, and bracketing was used to avoid assumptions or interpretations. Coding involved using line-by-line analysis to determine units of meaning that addressed either research question. When searching for themes, codes were grouped together into broader candidate themes in a ‘master table’ which highlighted the richness and diversity of the dataset. Quotes from specific participants were chosen due to their ability to capture the richness of the data set, highlight the relevant theme and address the research questions.

## Results

### Sample Characteristics

Participants mean age was 16.41 (*SD* = 1.02, min – max = 15.27 - 17.98). All participants except for one were attending CAMHS and all participants were White British. Further details can be found in Table 1.

**Table 1.** Participant Characteristics and Demographic Information (N = 9)

Sample characteristic	*N* (%)	*M* (min – Max; *SD*)
Age		16.41 (15.27-17.98; 1.02)
Gender		
Female	6 (66.7)	
Non-female	3 (33.3)	
Sexual orientation		
Heterosexual	3 (33.3)	
Not heterosexual	6 (66.7)	
Ethnicity		
White British	9 (100)	
Current voice-hearing		
Yes	8 (88.9)	
No	1 (11.1)	
Service attended		
CAMHS	8 (88.9)	
EIPS	1 (11.1)	
Current medication		
Yes	4 (44.4)	
No	5 (55.6)	
Psychological therapy†		
Yes	8 (88.9)	
No	1 (11.1)	

*Note.* CAMHS = Children and Adolescent Mental Health Services; EIPS = Early Intervention in Psychosis Services.

† This refers to having received any psychological therapy.

### Thematic Analysis

**Figure 2.**
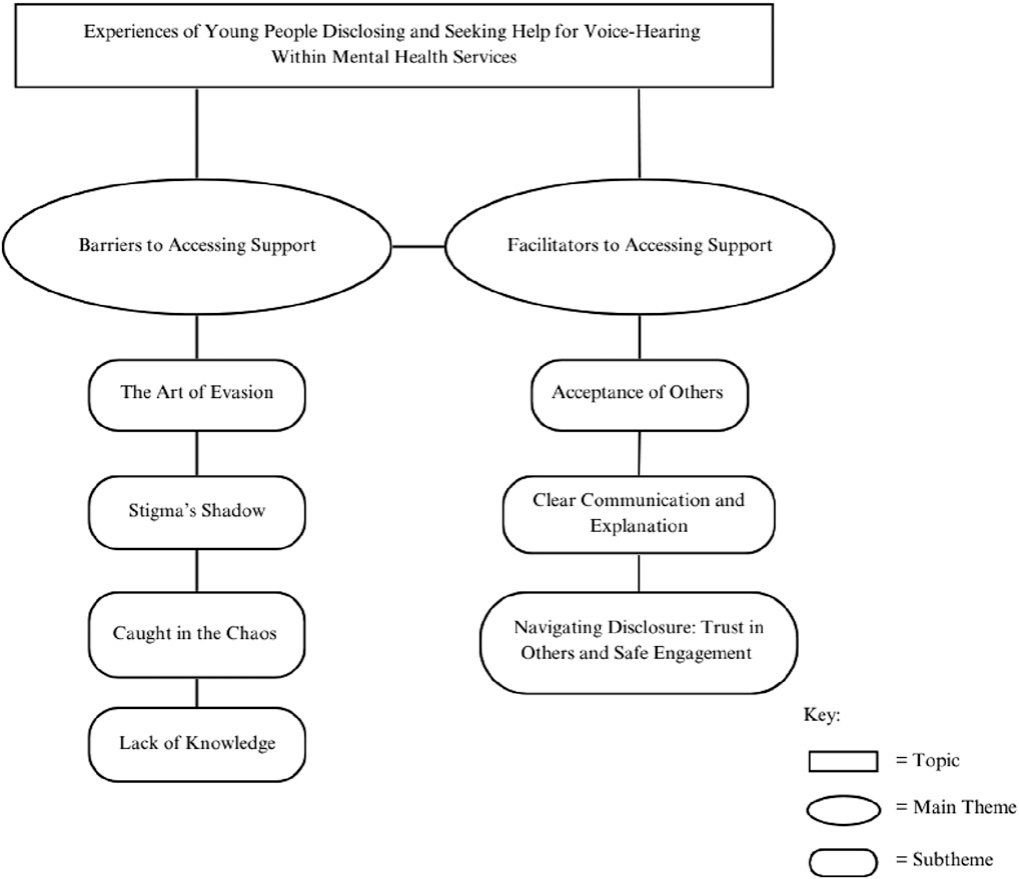
A Thematic Diagram Illustrating Themes, Subthemes and Their Relationships

Analysis identified two superordinate themes and their subthemes. Each is illustrated below. A thematic diagram illustrating each theme and their subthemes can be found in Figure 2.

### Barriers to Accessing Support

Participants experienced frustration, fear and anxiety during their disclosure of and help-seeking for voice-hearing. Within the main theme of barriers to help-seeking, the following subthemes were identified: participants often faced stigma from practitioners and wider society (Stigma’s Shadow) leading to avoidance of appointments (The Art of Evasion); many participants faced difficulties navigating systems and long wait times, delaying help-seeking (Caught in the Chaos); yet within services, participants encountered practitioners who seemed to have limited knowledge of voice-hearing, which participants felt contributed to mutual avoidance of this topic (Lack of Knowledge).

#### Stigma’s Shadow

During the process of disclosure and help-seeking, participants acknowledged the impact of negative judgement surrounding voice-hearing. The majority of participants (7/9) spoke of the impact that negative attitudes about voice-hearing in society and the media had on their feelings about disclosure. Charlie explained how this impacted his decision to not initially disclose his voice-hearing:

“when you say you hear voices, everyone just assumes you’re like dangerous and crazy... and I didn’t really want that association… when you say voices people think of like schizophrenia movies, where you just go around stabbing people or whatever… I didn’t want that” (Charlie)

This also affected participants who wanted to find information regarding voice-hearing. They expressed that searching online for information often led them towards stigmatising answers:

“I think the best thing they can do is sort of try to not stigmatise it and make it more acceptable and less demonised because all you hear about is worse case scenarios, TV and that stuff…. And it’s like you don’t hear any positives and if you do, you have to really search for it yourself. And if you do, it’s like finding a needle in a haystack or something” (Sam)

When participants entered services, nearly half (4/9) experienced judgement from practitioners. This experience often reinforced the external stigma they had experienced. Summer explains the feelings of shame or guilt she felt when discussing voice-hearing experiences:

“They sit there and they just look at you really funny throughout the whole meeting you know they give you really weird looks... they look at you like you should be put into a fucking insane asylum” (Summer)

#### The Art of Evasion

When considering accessing help, the feelings experienced by many participants (7/9) towards mental health services often led them to withhold their disclosure or avoid attending scheduled appointments:

“I always used to think it was a load of bollocks and it was like I don’t really want to go into. I felt like if I went there and said that [the voices], that would make me, I would have to spend more time there” (Georgie)

For some participants, these feelings were exacerbated when they felt practitioners were judgmental or pressurising. Emma explains how these experiences led her to avoid services or practitioners. This often facilitated a loop, with services failing to offer reassurance that they could reduce distress:

“Every time I knew I was going [to] CAMHS I was like “fuck sake I’m going [to] CAMHS. I’m not going.” And I’d try to make myself stop going even though I’d still get dragged there” (Emma)

#### Caught in the Chaos

All participants mentioned issues they had experienced whilst trying to access CAMHS, such as long waiting lists and fragmented support. This led to frustration from participants and their families, such as Jo’s mum who was unable to monitor the process fully and keep a record of Jo’s support history:

“She’s been in and out so many times it’s hard to keep track” (Jo’s Mum)

When they could monitor the process, participants mentioned the lengthy wait for support and attending multiple meetings with practitioners where they had to re-explain their circumstances. This further increased frustration and led Sam to consider disengaging from the process of disclosure:

“… do I even say anything cos it’s just gonna be more effort than it’s worth in the end which it wasn’t but that’s what it felt like at the time” (Sam)

This long process led Georgie to seek help privately with the knowledge that it would be quicker to access help and thereby reduce the impact of prolonged exposure to distress:

“The reason why we didn’t go to CAMHS the first time was… ‘cause I was so desperate and we knew that CAMHS had like months waiting lists. We were like we cannot do that.” (Georgie)

This was exacerbated by practitioners continually signposting elsewhere and leading to participants being placed upon further waiting lists. All participants were signposted from the service to which they initially disclosed their voice-hearing. In some cases, these onward referrals appeared to reflect structural limitations, such as fragmented care, or unclear service remit, as well as practitioner uncertainty or discomfort in responding to voice-hearing. These additional referrals provoked frustration for participants and help-seeking became more challenging:

“I had about four different meetings in the space of like a week or two with all different counsellors because they…looked through it with their team and were like yeh no, we can’t help you, we don’t know what’s wrong with you, oh try this person, oh go to this person…and it was like ‘are you serious?’” (Summer)

Additionally, participants felt as though the rigid and structured approach of services hindered their experience when discussing voice-hearing. They often wished for flexibility and more of a personalised approach that incorporated feedback (7/9). Participants expressed a need for variety in their support, extending beyond a ‘one size fits all’ approach:

“You know one week, I could just sit and need you to listen to me another week I could sit there and I need you to tell me what to do I need you to tell me how you can help me, you know or I need reassurance it’s gonna always be different, you just gotta keep asking, which a lot of people and a lot of places don’t do, once you tell them that first time how they can help, that’s it they’re stuck on that path they don’t drift from it you know, but to continuously ask… is this support still helping you …would you like us to try something different” (Summer)

#### Lack of Knowledge

When accessing support, participants often had little knowledge of their own needs in relation to managing distressing voice-hearing. This led more than half of the participants (5/9) to follow a practitioner’s guidance, assuming the practitioner would possess the knowledge they were seeking. This was also the case when they were turned away from services such as CAMHS, believing they did not need help:

“I sort of went oh well I’m not that- it’s not that bad” (Carson)

Yet, when accessing CAMHS many participants explained how they rarely spoke to a practitioner who could share knowledge on voice-hearing, and almost all participants (8/9) expressed frustration at services for being unable to provide explanations for their experiences:

“Like the fact I never really saw someone who really knew what they were talking about with the-[voice-hearing] was an issue” (Charlie)

Practitioners would frequently shift the focus of discussions away from voice-hearing. This led participants to believe that practitioners were avoiding discussions about voice-hearing due to discomfort or a lack of confidence:

Interviewer: “When you mentioned the voices and you said you want to talk about that this time, what happened?

Just same sort of things, like ‘oh no we won’t talk about that today’” (Linda)

This lack of practitioner knowledge left participants with concerns about the ability of practitioners to meet their needs. While some young people assumed professionals would have the answers, this seemed to reflect a broader uncertainty on both sides in making sense of their experiences. Without a single explanatory model for voice-hearing, practitioners appeared to approach help-seeking by ruling out various diagnostic possibilities, which some participants experienced as a box-ticking exercise. Summer felt that practitioners wanted to understand her experiences in relation to diagnostic criteria with no support plan outside of a diagnosis:

“The second you tell someone you hear voices they wanna put you in a box and they try and figure out which box you fit in…the worst thing is when you don’t fit into a box then they don’t know what to do with you” (Summer)

### Facilitators to Accessing Support

Participants appreciated feeling comfortable, safe and supported through their disclosure and help-seeking experiences. Trust in others, being given time to build rapport, and feeling genuinely heard (Navigating Disclosure: Trust in Others and Safe Engagement) helped create the conditions necessary for disclosure). Non-judgmental responses from others (Acceptance of Others) were imperative in facilitating self-acceptance and clear communication from services about the support they could offer (Clear Communication and Explanation) enabled continued engagement.

#### Navigating Disclosure: Trust in Others and Safe Engagement

Participants described a range of timeframes between the onset of voice-hearing to disclosure, varying from a week to several months or years. Trust in others played a key role in choosing whom to disclose to (8/9); some chose family, others chose their current practitioners in services and in schools, and one participant confided in friends. In some cases, the ‘trusted person’ accompanied them to appointments and helped them discuss their experiences until trust was established with practitioners:

“I think my first one, my mum might’ve come in and said.... for me that I heard voices.... I know the first time I went there was with my mum...I remember the woman came in. I kind of looked at her like “who the fuck are you?” ... And, um I'm pretty sure I turned around to my mum like “come in with me. Come in with me” ... It’s like my first ever appointment I would’ve took her in. Like I know that 100%. I wouldn’t have gone in there by myself” (Emma)

When attending appointments, participants valued practitioners who allowed time for trust and rapport to be built. This promoted an environment for them to feel safe and supported in discussing their experiences:

“He was asking details for things I wasn’t ready to talk about especially with him. Because he was a man and he, had like met him once. So I didn’t like trust him at all and then I wasn’t comfortable talking about it… and then I got [a different practitioner] and then it was much better. Cause we like got to know each other…it was like we didn’t go into heavy stuff until I could deal with it.” (Sam)

Participants appreciated practitioners who demonstrated care and understanding when working together. For many (7/9), this included the practitioner listening. When practitioners did not listen, this often hindered the development of trust and rapport and could lead to inaccurate assumptions:

“I think that was the first thing that really put me off CAMHS like properly made me think that CAMHS are a bit shit in a way… it was like, I don’t know, they just. They don’t listen to like everything I guess. I guess they kind of like listen to what they want to listen to and put it down to what they wanted to.” (Emma)

For participants who felt as though their practitioner demonstrated patience, tailored support and actively listened to them, this was highly valued:

“Psychologists like [practitioner], are amazing cause I know he’s not there for money. He’s passionate about his job, he comes up with strategies to help me and stuff so. It’s quite nice to be fair” (Finley)

When support was tailored towards participants, they felt it demonstrated they were being cared for by services, yet all participants felt uncared for at times during their help-seeking. Participants sometimes felt like they were an individual who needed to be ‘fixed’ or that their care was impersonal with practitioners feeling ‘robotic’ during assessments:

“I feel like every time I went to go see her it was always the same thing... Like, I don’t know. She was just really like robotic. Like, she would just ask questions and I would answer them, and she would write them down. There was no like, yeah, I don’t know. I just really didn’t like her.” (Jo)

Participants wanted practitioners to listen to their problems. For some (5/9), listening and trying to understand their situation was important regardless of the knowledge practitioners possessed:

“you talking and listening, for that half an hour or even ten minutes of you just shutting up and listening, so fucking helpful” (Summer)

Finally, when discussing the care they received, participants expressed a longing for more. Often, this included young people wanting to talk more about their voice-hearing, and discussing these experiences was often reported as the most valued support they received:

Interviewer: “What do you value most from the support you received from CAMHS for the voice-hearing experience in specific?

Probably the psychologist who, like, actually helped me to properly talk about it.” (Charlie)

Whilst most participants wanted to discuss voice-hearing in sessions (8/9), this feeling varied throughout their support, changing as rapport was built. Initially, Finley felt reserved regarding discussing his voice-hearing as it felt “too personal”, but with time he realised that discussing these experiences built his confidence. At other times, participants had priorities beyond voice-hearing and wanted to discuss other issues in sessions (6/9). Participants valued having the option to discuss other issues with the awareness that a practitioner would listen if they wished to discuss their voice-hearing:

“I tell her everything, and she was like “Do you wanna talk about it?” and I was like “No, not really”. (Georgie)

#### Acceptance of Others

The reactions of other people to discussion of voice-hearing impacted participants’ self-perception. Most of the participants (8/9) expressed gratitude to non-judgemental practitioners and emphasised the importance of a non-judgmental approach when discussing voice-hearing:

“I felt like I was saying really insane things but... people were just accepting what I was saying understanding it without criticising or making me feel like it was something wrong with me” (Carson)

Some participants (5/9) valued voice-hearing being treated as a symptom of mental health conditions, and this helped to counteract the stigma from wider society and facilitated an accepting environment:

“Reassure them that they’re not crazy and they’re not losing their minds, you know, what they are experiencing is normal and that they’re not the only one” (Summer)

Acceptance from others generated a sense of self-acceptance which, for many (7/9), improved their ability to cope with voice-hearing and discuss it openly, leading to improved engagement with services:

“When I kind of remind myself that you know what happens is going to happen and you know, there's nothing I can do about that, then I kind of like calm myself down. And then I like, I can kind of drown it out.” (Georgie)

#### Clear Communication and Explanation

When participants were using services, clarity on treatment plans, decision-making and feedback from participants was important. Most (8/9) received resources on voice-hearing such as helplines and leaflets, but fewer had services fully explained to them including how often they will meet with a practitioner, what the practitioner will assist with and collaborative goal setting for future work (2/9). Carson appreciated how CAMHS outlined their support and timeline before their next meeting, providing context and setting expectations:

“I met one of the CAMHS workers [name of worker], and, she explained to me what CAMHS was and said this is what we wanna help you with - we’ll meet up with you in a few weeks’ time” (Carson)

Participants expressed the wish for more coping and prevention strategies when planning the management of distressing voice-hearing. For most participants (8/9), they explained how this would be a priority in therapy going forward, but some also explained how they found prevention helpful and longed for knowledge of this sooner:

“Like, I never used to know my triggers as (inaudible) and it’s like its only [practitioner] who even mentioned to me like, try and notice like your triggers. Try and notice what happens beforehand” (Emma)

Participants valued being asked for their input in treatment. This promoted an environment that felt comfortable and allowed them to re-claim their experience:

“They are quite good people and if they do listen to what people have to say and if they listen to all our feedback which they do then it makes them better person.” (Finley)

Finley, who attended EIPS, described the positive aspects of his care. He felt strongly about his care in EIPS and described the ‘fantastic’ support he was able to receive and how this enhanced his ability to discuss his voice-hearing after leaving their care:

“I wouldn’t say I had any like bad experiences of it. So when the care was in place it was in place. It was all set out and all planned it was really good care. I received several (inaudible) they were friendly towards me. They knew how to cope. They knew how to react with me so I don’t have any bad points about that so yeah. The care I received was very good” (Finley)

## Discussion

The aim of this study was to investigate young people’s experiences of disclosure of distressing voice-hearing and help-seeking within mental health services. Two superordinate themes were identified following the analysis of interviews with nine young people. The themes addressed the barriers of disclosure and help-seeking, alongside the importance of trusted relationships and feeling safe to open up and engage with services. Within these themes, suggestions were offered for the help that would ideally have been received.

Some of the barriers to disclosure and help-seeking were not unique to voice-hearing, including systemic barriers such as long waiting times and external societal stigma (Bogen-Johnston et al., 2019; Vilhauer, 2015; Yttri et al., 2020). However, others related more specifically to voice-hearing including stigma from practitioners, fragmented support and knowledge gaps on voice-hearing displayed by practitioners. These findings corroborated research by Kapur et al. (2014) with practitioners’ lack of knowledge and confidence in delivering support for voice-hearing experiences negatively impacting participants’ help-seeking. Importantly this finding may also reflect the inherent complexity of voice-hearing as a phenomenon that lacks a unified explanatory framework. Both young people and practitioners appeared to encounter challenges in understanding and contextualising these experiences, indicating the relevance of developmentally informed, formulation-based approaches that enable shared meaning-making within clinical settings.

Some of the helpful responses identified by participants were not unique to voice-hearing, including allowing time for trust and rapport to build in sessions and clear communication from services enabling smooth disclosure and help-seeking experiences (Bogen-Johnston et al., 2019; Kapur et al., 2014). Indeed, the young people suggested that practitioners may benefit from drawing upon some of the core skills required by mental health practitioners to build rapport, including patience, non-judgement and self-awareness (Rogers, 1957). In this sense, voice-hearing experiences may benefit from being de-catastrophised and ‘normalised’, and being positioned as part of a broader continuum of mental health experiences rather than as an extraordinary or inherently pathological phenomena. This would require practitioners to be more knowledgeable about voice-hearing experiences, including recognition of its transdiagnostic nature and the potential for voices to convey meaningful insights into underlying psychological needs or difficulties. Information from the literature on emerging interventions for the treatment of distressing voice-hearing in young people could also be shared (Hayward et al., 2022; Maijer et al., 2020), with an emphasis placed upon the value and use of generic therapeutic skills when delivering these interventions.

## Clinical Implications

These findings indicate the value of establishing safety and trust through active listening, validation, and clear communication, in supporting disclosure and engagement among young people who hear voices. This aligns with national guidance emphasising trauma-informed care (Department of Health and Social Care, 2022), youth-friendly relational approaches (Office for Health Improvement and Disparities, 2023) and developmentally appropriate, person-centred communication and continuity in young people’s mental health care (National Institute for Health and Care Excellence, 2021).

Additionally, findings emphasise the importance of education and support for practitioners to increase both their knowledge of voice-hearing and their confidence to facilitate conversations with young people about these experiences. Prior research has emphasised the value of using structured assessments to guide these initial conversations (Bogen-Johnston et al., 2019), as this may address the uncertainty reported by many practitioners about how to explore voice-hearing experiences effectively (Byrne et al., 2020). Recent findings also suggest that access to supportive assessment and psychoeducational materials may help CAMHS clinicians engage more confidently in these discussions (Rammou et al., 2023b). Thereafter, subsequent conversations may need to be facilitated by practitioners who have been trained in the delivery of brief interventions designed specifically for young people who are distressed by hearing voices and similar experiences. Two interventions are currently being evaluated that may add value in this respect: the CHUSE study (Parry, nd) (ISRCTN12245618) – a compassion-focused intervention for young people with unusual sensory experiences; and the ECHOES study (Hayward, nd) (ISRCTN16395888) – a coping intervention being delivered within schools and aiming to facilitate early access to helpful conversations within a setting that is familiar to young people. Both trials emphasise the importance of discussions about voice-hearing being facilitated by people outside of clinical settings, through the provision of educational opportunities for parents (CHUSE and ECHOES) and school staff (ECHOES). Peer support may also facilitate disclosure of and coping with voice-hearing (Newton et al., 2007), and practitioners may benefit from familiarising themselves with and signposting young people to peer-support resources such as Voice Collective, a national project that supports young people who hear voices (https://www.voicecollective.co.uk). Together, these interventions and resources underscore the importance of multi-layered support strategies that extend beyond clinical settings, fostering open conversations, early intervention, and community-based support networks. Finally, involving young people in co-producing training and service design may enhance the relevance and accessibility of care by promoting empowerment and engagement, and ensuring services are responsive to young people’s needs (Norton, 2021; Viksveen et al., 2024).

## Limitations

This study has several limitations. Firstly, using a transdiagnostic approach led to the exclusion of much literature due to unclear distinctions between psychotic experiences, hallucinations and voice-hearing. This highlights the need for future research to report findings with more clarity, identifying which experiences researchers are referencing rather than using the terms “psychotic experiences”, “hallucinations” and “voice-hearing” to mean one collective experience (de Leede-Smith & Barkus, 2013). Secondly, all participants identified as White British, thereby limiting the transferability of the findings; future studies could investigate the influence of ethnicity and cultural context on disclosure and help-seeking for voice-hearing among young people, as stigma surrounding mental health care (Ahad et al., 2023) and the cultural sense-making of voice-hearing experiences (Larøi et al., 2014) can vary significantly. Future research could also explore how help-seeking is shaped by cultural beliefs, stigma, and structural barriers in racially minoritised groups, recognising that these factors may uniquely influence access to and engagement with mental health services, including levels of trust in providers and perceived cultural alignment (Jacobs & Pentaris, 2021). This may provide insight into experiences not represented in the current study. Thirdly, the retrospective nature of the interviews may have resulted in inconsistencies in the recollections of participants. Finally, this study only offers insights from the experiences of young people who were engaged with mental health services and who had disclosed their experiences to clinicians, even though they may not have received support for distressing voice-hearing. Notably, the sample did not include participants younger than 15 years, despite evidence that younger children can experience voice-hearing and access services. This represents an important gap, as younger age groups may encounter distinct barriers to help-seeking and disclosure not captured in the present study. Future research should aim to include those who have not accessed mental health services or who fall below service thresholds, as these groups may encounter different barriers to disclosure and support that are not reflected in the current findings.

## Supplemental Material

Supplemental Material - “We Are Humans, and We Are People” - A Thematic Analysis Exploring the Disclosure and Help-Seeking Experiences of Young People Who Experience Voice-Hearing Within Mental Health Services in the UK Supplemental Material for “We Are Humans, and We Are People” - A Thematic Analysis Exploring the Disclosure and Help-Seeking Experiences of Young People Who Experience Voice-Hearing Within Mental Health Services in the UK by Megan Ellis, Mark Hayward, Clio Berry, David Fowler, Aikaterini Rammou in Clinical Child Psychology and Psychiatry.

## Data Availability

The data that support the findings of this study are available from the corresponding author on reasonable request. The data are not publicly available due to privacy or ethical restrictions.*
