# Supplementary material for: “We Are Humans, and We Are People” - A Thematic Analysis Exploring the Disclosure and Help-Seeking Experiences of Young People Who Experience Voice-Hearing Within Mental Health Services in the UK
Source: Clin Child Psychol Psychiatry. 2025 Nov 25;31(2):744–63. doi: 10.1177/13591045251400393 (PMC12992644; doi:10.1177/13591045251400393)
Supplement: Supplemental Material - “We Are Humans, and We Are People” - A Thematic Analysis Exploring the Disclosure and Help-Seeking Experiences of Young People Who Experience Voice-Hearing Within Mental Health Services in the UK [file sj-pdf-1-ccp-10.1177_13591045251400393.pdf]

Semi-structured Interview Guide

"To begin with, I was wondering if you could tell me a few things about the **time** the voices started and what happened after that."

<u>General Questions about the voice-hearing experience</u>

1) When did the voice(s) start? What was happening in your life during that period?

2) Do you respond to the voice(s)?

If NO: what do you do when the voice(s) talk?

If YES: How do you respond to the voice(s)?

<u>Disclosure</u>

3) When you first experienced voice(s), did you tell other people about your experience?

If YES: who did you tell? Why did you choose this(these) person(s)?

4) Now, do you feel able to talk to people about the voice(s) you hear?

If YES: whom can you talk to?

If NO: why do you think it is difficult for you to talk about this experience?

<u>Help-seeking</u>

5) Have you sought support through mental health services or other voluntary/private/school services in relation to your experiences of hearing voice(s)?

If YES: Which services have you sought help from?

If NO: Is there a reason or reasons you decided not to seek support for your voice-hearing from services?

[If they haven't asked for help with regards to voice-hearing from NHS services consider skipping to <u>Question 21</u>]

6) Have you sought support elsewhere?

"Thank you for sharing this. Now, I will ask you a few questions about your experiences of accessing mental health services in relation to your voice-hearing experience."

<u>Care Pathway in mental health services</u>

7) From your experience, what steps did you take to receive support from CAMHS for your voice-hearing?

8) When you first contacted mental health services, were hearing voices your (or one of your) main problem(s)? What were your main problems/difficulties?

9) From your experience, what/who led you to seek support from CAMHS/EIP services? How?

10) From your experience, did anything or anyone help you to find and access support from CAMHS/EIP services in relation to your voice-hearing?

If YES: What and Who helped?

<u>Barriers to accessing support</u>

11) Did you have any difficulties to receive support from CAMHS/EIP in relation to your voice-hearing?

If YES: What were these difficulties?

<u>Clinicians' response to disclosure</u>

12) From your experience, how did the clinician(s) respond when you first disclosed your voice-hearing experience?

13) How did they end your conversation and your meeting?

Probes: Did they offer any follow-up appointment to pick up the conversation then?

<u>Clinician's Immediate Support</u>

14) From your experience, what short-term/immediate support did your CAMHS/EIP clinician offer you after disclosing your voice-hearing experiences?

<u>Long-Term Support/Treatment</u>

15) From your experience, what long-term support or treatment was offered from CAMHS/EIP with regards to your voice-hearing?

Probes: Any psychological/talking therapy? Any family meetings with a clinician? Any medication prescribed?

16) Did you receive any of the long-term support offered?

IF YES to any of this, ask more details

17) Did you create any plan to help manage distressing voice-experience? What did this entail?

18) Did CAMHS/EIP recommend that you access or refer you to any other services?

<u>Positive aspects of current care/support</u>

19) What do you value most from the support you've received by CAMHS/EIP services for your voice(s) hearing experience?

Probes: What did you find most helpful so far? (e.g. talking to a clinician, therapy, medication, support groups?)

<u>Suggested changes to current care/support</u>

20) Is there anything you find unhelpful about the support you're receiving from CAMHS/EIP services?

Probes: What is that? Why do you find it unhelpful?

21) Is there anything else that you think needs to be changed in the way you are receiving support at the moment?

Probes: What can the clinicians do better? Any changes in structure of the service? Any changes in the service processes? Any changes in the support provided?

Additional support suggestions

22) What do you think mental health services can do to further support young people who hear voices?

Psychological Therapy

23) What would a psychological therapy look like to be most beneficial for young people?

24) What would be your priorities in a psychological therapy?

Probe: What would you rate as most important for a psychological therapy?

25) Would distressing voice-hearing and how to manage it be your priority in therapy (*note: for both young people receiving therapy and those who don't*)?

General/End

26) Is there anything else you would like to tell me that I haven't asked you about?

27) Do you have any questions for me about anything that we've talked about?

28) How did you find the interview today? What are your plans for the rest of the day?

Thank you for your time. This is the end of the interview.
